# Influence of micro-milling machining parameters on residual stresses in alumina bioceramics-a three-dimensional finite element simulation study

**DOI:** 10.1371/journal.pone.0313588

**Published:** 2024-11-14

**Authors:** Zhen Wang, Yanan Sun

**Affiliations:** 1 College of Mechanical and Electronic Engineering, Shandong University of Science and Technology, Qingdao, Shandong, China; 2 State Key Laboratory of Crane Technology, Yanshan University, Qinhuangdao, Hebei, China; 3 Guangdong Provincial Key Laboratory of Minimally Invasive Surgical Instruments and Manufacturing Technology, Guangdong University of Technology, Guangzhou, Guangdong, China; 4 School of Information Science and Engineering, Yanshan University, Qinhuangdao, Hebei, China; TU Dublin Blanchardstown Campus: Technological University Dublin - Blanchardstown Campus, IRELAND

## Abstract

The formation and distribution of residual stress during the micro-milling process significantly affect the crack resistance and service life of alumina bioceramics. This study aims to optimize the surface residual stress distribution by adjusting machining parameters, thereby improving the machining quality of alumina ceramics. A three-dimensional finite element model of alumina bioceramics was developed, and numerical simulations were conducted to analyze the effects of feed per tooth, cutting depth, and spindle speed on temperature and residual stress. The study further explores the patterns of residual stress variation. The results show that both surface temperature and residual tensile stress exhibit systematic trends with parameter changes. Specifically, surface residual tensile stress increases with cutting depth initially but decreases sharply once the cutting depth exceeds 25 μm. Residual tensile stress increases with spindle speed, reaching its peak at 21,000 r/min before stabilizing. Additionally, the residual tensile stress rises with feed per tooth at first but gradually declines when the value exceeds 25 μm/z. This research reveals the mechanisms by which micro-milling parameters influence surface temperature and residual stress in alumina bioceramics, providing theoretical guidance for optimizing micro-milling processes. The findings can also be extended to the micro-milling of other hard-to-machine materials, offering broad engineering application potential.

## 1 Introduction

Micro-milling, as a high-precision machining technology, is widely used in the field of precision manufacturing. At the micron scale, the cutting characteristics and surface integrity of materials directly influence machining quality and product performance [1]. In micro-milling, machining parameters such as feed rate, cutting depth, and spindle speed not only affect material removal efficiency and surface quality but also play a decisive role in the formation and distribution of residual stress [2,3]. Residual stress refers to the internal stress remaining in a material after the removal of external forces, which can significantly impact fatigue performance, crack resistance, and dimensional stability [4]. Alumina bioceramics, due to their excellent biocompatibility and mechanical properties, have become ideal materials for medical implants and other high-performance applications [5,6]. In the case of alumina bioceramics, appropriate residual compressive stress helps enhance crack resistance, whereas excessive residual tensile stress can lead to crack initiation and propagation, thereby reducing the material’s service life and reliability. Therefore, optimizing micro-milling parameters to control the distribution of residual stress has become a key research focus for improving the machining quality of alumina bioceramics.

Finite element simulation, as a powerful numerical analysis tool, has been widely applied in the study of material processing [7,8]. By developing a three-dimensional finite element model, it is possible to simulate the cutting forces and temperature fields during micro-milling, providing a deeper understanding of how different machining parameters affect these variables [9]. Many researchers have combined finite element simulations with experimental methods to investigate surface residual stress, revealing the mechanisms by which machining parameters influence residual stress in materials [10,11]. However, most of these studies focus on residual stress in the machined surfaces of metallic materials, while limited research has been conducted on residual stress in hard and brittle ceramic materials. To address this gap, this study, based on the characteristics of the micro-milling process, develops a three-dimensional finite element model of alumina bioceramics. Through numerical simulations, the effects of feed per tooth, cutting depth, and spindle speed on the residual stress of alumina bioceramics are analyzed. Additionally, the mechanisms of residual stress formation and its evolution are explored. This study provides theoretical guidance for optimizing the micro-milling process and promotes the advancement of high-precision machining technologies.

The structure of this study is divided into six sections:(1) Introduction: Clarifies the research background, objectives, and significance;(2) Literature Review: Summarizes the research progress on machining of bioceramic materials and residual stress in cutting processes;(3) Research Design: Describes the methods for constructing the finite element simulation model and explains the constitutive model of alumina bioceramics;(4) Results and Analysis: Analyzes the simulation results of the milling temperature field and residual stress;(5) Discussion: Interprets the significance of the findings, including contributions and limitation;(6) Conclusion: Summarizes the research outcomes and proposes future research directions.

## 2 Literature review

### 2.1 Progress in processing research of bioceramic materials

As an emerging biomaterial, bioceramics have attracted extensive attention in recent years due to their excellent biocompatibility, mechanical properties, and bioactivity. The main types of bioceramics include oxide ceramics, phosphate ceramics, and glass ceramics. Among them, oxide ceramics, such as zirconia and alumina, are widely used in bone repair and dental restoration due to their high strength and superior biocompatibility [12]. Phosphate ceramics, such as hydroxyapatite and tricalcium phosphate, are commonly employed in bone grafts and defect repairs because their chemical composition closely resembles that of human bone and they exhibit favorable biodegradability [13]. Glass ceramics, with their excellent mechanical properties and bioactivity, are used in bone replacement materials and dental restorations [14]. The machining technology of bioceramic materials directly affects their clinical performance and effectiveness. Therefore, conducting in-depth research on the machining techniques and advancements in bioceramic materials holds significant practical importance.

Micro-milling is a machining method that removes material using micron-scale tools. Its advantages in the machining of bioceramics are reflected in several key aspects: First, high-precision machining. Micro-milling technology can achieve micron-level precision, which is crucial for machining complex shapes and small features in bioceramic materials. Gao et al. [15] found that by optimizing micro-milling parameters, sub-micron feature machining can be achieved on bioceramic surfaces, meeting the stringent requirements of high-precision medical devices. Second, low cutting forces. Due to the small size of the cutting tools, the forces generated during micro-milling are relatively low, which helps minimize damage and prevent crack formation in bioceramic materials. Liu et al. [16] experimentally confirmed the advantages of low cutting forces in micro-milling, demonstrating that these forces significantly reduce surface cracks and internal defects, thereby improving machining quality. Third, high efficiency. Micro-milling enables efficient material removal, reducing machining time and improving production efficiency. Zhang et al. [17] optimized the cutting parameters in their study, achieving more than a 20% increase in machining efficiency for bioceramic materials while maintaining high surface quality. inally, superior surface quality. Micro-milling can produce excellent surface finishes, minimizing the need for additional surface treatments. Chen et al. [18] reported that with appropriate micro-milling techniques and parameters, extremely low surface roughness could be achieved on bioceramics, eliminating or reducing the need for subsequent polishing processes, thus saving time and costs.

3D printing technology has shown tremendous potential in the manufacturing of bioceramic materials. It allows precise control over the shape and internal structure of materials based on computer-aided design (CAD) models, enabling the personalized production of bioceramic components. This technology not only enhances the precision of implants but also shortens manufacturing cycles and reduces costs. Wang et al. [19] fabricated alumina implants with complex geometries using 3D printing, demonstrating that these implants exhibited excellent mechanical properties and biocompatibility. Additionally, Li et al. [20] developed a novel 3D-printed alumina/hydroxyapatite composite. By optimizing printing parameters and material ratios, they significantly enhanced the mechanical strength and bioactivity of the composite.

Laser sintering is another advanced technique that uses laser energy to fuse and solidify powdered materials, precisely controlling the temperature and duration of the sintering process to produce high-performance bioceramics. Chen et al. [21] successfully prepared high-purity alumina ceramics using laser sintering, reporting outstanding mechanical strength and corrosion resistance. Similarly, Liu et al. [22] studied the sintering behavior of alumina particles during laser sintering and found that optimized sintering parameters significantly improved the material’s density and mechanical properties.

In summary, research on the processing technologies of bioceramics has made remarkable progress over the past few years. With technological advancements and the growing demand for biomedical materials, future research could focus on improving material degradation rates, enhancing bioactivity, and developing bioceramics with antibacterial and anti-infection properties. These advancements will further broaden the application prospects of bioceramic materials.

### 2.2 Research progress on residual stress in cutting machining

Residual stress in machining refers to the stress induced on the surface and within the interior of a workpiece due to the interaction between the tool and the workpiece during the cutting process. This stress remains in the material even after machining is completed and is not fully released. Residual stress can significantly affect the performance and service life of the machined component. Researchers have focused on understanding the impact of thermo-mechanical coupling during the cutting process on residual stress distribution. During machining, friction and deformation between the tool and the workpiece generate significant heat, leading to thermal expansion and possible phase transformations in the material, both of which influence the residual stress distribution. In recent years, advanced numerical simulation techniques, such as finite element analysis (FEA), have been employed to simulate the thermo-mechanical interactions during cutting. These simulations not only improve our understanding of the machining process but also provide a theoretical foundation for optimizing machining parameters. Ding et al. [23] conducted a detailed analysis of residual stress using a thermo-mechanical coupling model and found that cooling conditions and cutting speed significantly affect the stress distribution. Similarly, Smith et al. [24] used a comparable approach, further confirming the variations in residual stress across different materials under varying cooling conditions.

The optimization of cutting parameters is another critical area of research for reducing residual stress. Key cutting parameters include cutting speed, feed rate, depth of cut, and tool geometry, all of which directly influence cutting forces and heat generation, thereby affecting the formation of residual stress. In recent years, multi-factor optimization methods based on experiments and simulations have been widely applied. Zhang et al. [25] systematically optimized cutting parameters using design of experiments and response surface methodology, significantly reducing surface residual stress. Similarly, Lee et al. [26] effectively minimized residual stress during cutting by improving tool geometry and optimizing the feed rate.

Research on material properties has also become a major focus in the study of machining-induced residual stress. Different materials exhibit varied stress responses during cutting, making it essential to investigate residual stress formation for specific materials. With the emergence of advanced materials, such as high-strength steel, titanium alloys, and composites, researchers have conducted in-depth studies on the residual stress in these new materials. Li et al. [27] systematically analyzed the residual stress in titanium alloys during cutting and found that the high-temperature stability of titanium alloys plays a crucial role in stress formation. In another study, Chen et al. [28] investigated the residual stress characteristics in high-strength steel, revealing that appropriate cutting speeds and cooling conditions can significantly reduce residual stress.

In conclusion, research on residual stress in machining has made significant progress through the application of thermo-mechanical coupling models, optimization of cutting parameters, and studies on new material properties. However, most of this work has focused on residual stress in machined metallic surfaces, while the residual stress in the machined surfaces of hard and brittle ceramic materials requires further investigation. This is precisely the focus of the present study.

## 3 Research design

### 3.1 Construction of finite element simulation model

In this study, a milling temperature field model was constructed using a milling tool with a length of 2 mm, a diameter of 1 mm, and a thickness of 0.2 mm, along with a workpiece model measuring 3 mm × 2 mm × 1 mm. The model is based on standard parameters widely adopted in previous studies [29,30]. These parameters have been validated to effectively capture the thermo-mechanical coupling and stress distribution characteristics of the micro-milling process, ensuring that the simulation results align with real machining conditions and possess high reliability and reference value. The workpiece was meshed according to the standard criteria for element quality, including aspect ratio, skewness, and warping factor. The size of the mesh elements for the workpiece was set to 0.01 mm to ensure precise simulation results.

The setup steps for constructing 3D micro milling using Ansys/ls dyna finite element software are as follows: Firstly, the milling cutter and workpiece adopt solid164 solid elements. Then, the thermal performance parameters, thermal boundary conditions, and thermal contact parameters of the aluminum bioceramics are defined and solved in the ls dyna solver in the form of a k file. The solid164 solid element can effectively handle the thermal coupling, large material deformation, and contact problems involved in the 3D micro milling process, ensuring that the simulation can accurately reflect the distribution of stress and temperature. Secondly, treat the milling cutter as a rigid body and constrain its rotational degrees of freedom in the x and y directions and translational degrees of freedom in the y and z directions. Meanwhile, set the thermal boundary parameters, as shown in Table 1. The rigid body assumption of milling cutters is based on their high hardness and stability, and their deformation can be ignored during micro milling. Therefore, this simplification not only helps to reduce computational complexity, but also ensures that simulation accuracy remains stable within a reasonable range. The rigid body assumption ignores the small deformations that milling cutters may undergo in high stress or high temperature environments, which may have a slight impact on the accurate simulation of local stress and contact forces. This influence is mainly reflected in the contact mechanical characteristics between the cutting tool and the workpiece during the milling process, which may lead to slight deviations between the simulated results of stress concentration areas and the actual situation. But the focus of the research is on the temperature field and residual stress variation of alumina bioceramics, and these deviations have little impact on the overall results of this study. Therefore, although this assumption has limitations under extreme conditions, it does not have a substantial impact on the overall trend and conclusions of the simulation results, and the research results still have high accuracy and reliability. Finally, select surface to surface eroding (ESTS) contact as the contact method between the workpiece and the milling cutter, and set the gmax value to 1.

**Table 1:** Thermal properties of alumina bioceramics.

Thermal Property	Value
Density	3.8 g/cm^3^
Specific Heat Capacity	0.9 J/g·K
Thermal Expansion Coefficient	9.5×10^−6^ K^-1^
Thermal Conductivity	20W/m·K
Elastic Modulus	380Gpa

To simulate the residual stress on the workpiece surface using Ansys/LS-DYNA based on the milling temperature field model, follow these detailed steps: Begin by using the transient temperature field simulation results as the foundation. In LS-DYNA, modify the analysis type to thermo-mechanical coupling by setting the CONTROL_SOLUTION keyword to 2. Set the simulation time to 0.02 s to capture the transient thermal-mechanical behavior accurately. Perform the 3D micro-milling finite element simulation until the system reaches a steady-state condition. After completing the milling process, withdraw the milling tool from the workpiece surface. Remove all external constraints from the workpiece to allow the material to elastically recover and cool down in the absence of external loads. Obtain the Residual Stress Distribution.

### 3.2 The constitutive model of alumina bioceramics

This study used the Johnson Holmquist (JH-2) model to evaluate the failure behavior of materials, and the equivalent stress of alumina bioceramics is:

σ*=σi*‐D(σi*‐σf*) (1)

Among them, the nominal equivalent stress σ* = σ/σHEL, where σ is the actual equivalent stress, σHEL is the equivalent stress of alumina bioceramics at the elastic limit, and σ is

$$\sigma =\sqrt{\frac{1}{2}[{\left(\sigma_{x}-\sigma_{y}\right)}^{2}+{\left(\sigma_{x}-\sigma_{z}\right)}^{2}+{\left(\sigma_{y}-\sigma_{z}\right)}^{2}+6({\tau_{xy}}^{2}+{\tau_{xz}}^{2}+{\tau_{yz}}^{2})]}$$ (2)

Among them, σx, σy and σz are the normal stresses in three directions, and τxy, τxz and τyz are the shear stresses in three directions, respectively. In Formula (1), σi*and σf* represent the strength of the material before failure and the nominal fracture strength of the material, respectively;

σi*=A(P*+T*)N(1+Clinε*) (3)

σf*=B(P*)M(1+Clinε*) (4)

Among them, A is the strength coefficient, B is the fracture strength coefficient, T * is the maximum tensile fracture strength, and P * is the pressure value. Meanwhile, we defined the failure parameter D in the JH-2 model of alumina bioceramics:

$$D=\sum \frac{\Delta \varepsilon^{P}}{{\varepsilon_{f}}^{p}}$$ (5)

$${\varepsilon_{f}}^{p}=D1(P*+T*)D2$$ (6)

Among them, Δε^P^ is the shaping strain, ε_f_^p^ is the shaping failure strain, and the failure of the general unit occurs when the failure parameter D = 1.

## 4 Research results and analysis

### 4.1 Analysis of milling temperature field model results

In this study, the 3D micro-milling finite element model was configured with a spindle speed of 12,000 r/min, a cutting depth of 25 μm, and a feed per tooth of 25 μm/z to determine the temperature distribution in alumina bioceramics. We found the temperature in the alumina bioceramics is primarily concentrated beneath the tool tip and in its immediate vicinity. The temperature gradient increases as the material gets closer to the milling tool, indicating the poor thermal conductivity of alumina bioceramics. When the milling tool moves away from the material’s edge, the heat begins to spread outward, but only over a shallow depth, with the diffusion depth not exceeding 0.3 mm throughout the milling process. This phenomenon arises from the friction between the tool and the workpiece and the material deformation during machining, which generates significant heat concentrated in the cutting zone, causing a marked rise in temperature. However, due to the limited thermal conductivity of alumina bioceramics, the heat does not diffuse rapidly into distant areas, and the high-temperature zone remains localized near the tool. Consequently, the temperature gradient decreases sharply with increasing distance from the milling tool, indicating a shallow heat diffusion depth within the material.

This temperature distribution plays a critical role in the formation of residual stress. The heat concentration in the cutting zone causes localized thermal expansion, and as the material cools, the rapid cooling in these regions, compared to the surrounding cooler areas, generates significant thermal stress. This thermal stress becomes a primary source of residual stress. Due to the non-uniform heat distribution, high residual tensile stress tends to accumulate near the milling path, especially along the tool’s cutting trajectory. This observation highlights the close relationship between high-temperature zones and residual stress distribution. The elevated temperature in the cutting region not only affects the machining temperature but also plays a crucial role in the development of residual stress, further underscoring the importance of temperature control in micro-milling processes.

#### 4.1.1 The influence of cutting depth on the temperature of alumina bioceramics

**Fig 1:**
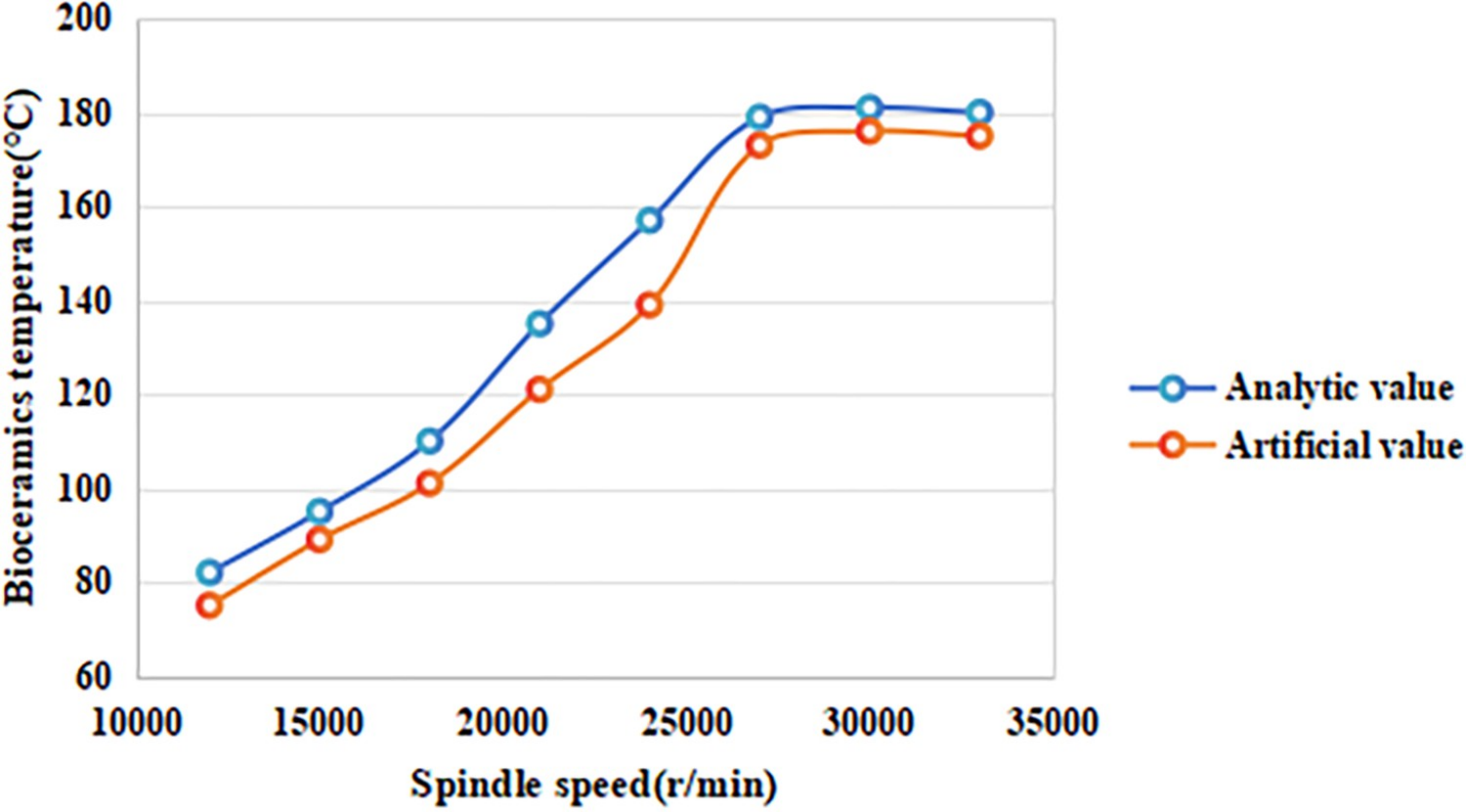
The effect of cutting depth on the maximum surface temperature of alumina bioceramics.

In the micro-milling temperature field simulation, the spindle speed was kept constant at 18,000 r/min with a feed per tooth of 25 μm/z, while the cutting depths were varied at 10 μm, 15 μm, 20 μm, 25 μm, 30 μm, 35 μm, 40 μm, and 45 μm to investigate the effect of cutting depth on the maximum surface temperature of alumina bioceramics. The results are shown in Fig 1. When the cutting depth is less than 25 μm, the surface temperature of the alumina bioceramics decreases gradually. This occurs because smaller cutting depths reduce the contact area between the tool and the workpiece, resulting in less frictional heat generation. In micro-milling, frictional force is positively correlated with the contact area, so reducing the area directly limits friction and heat production. Although the thermal conductivity of alumina bioceramics is relatively low, the heat generated at shallower depths is still limited enough for the workpiece to dissipate the heat efficiently to surrounding areas through localized thermal diffusion. A shallower cutting depth also means a shorter heat diffusion path and a smaller thermal gradient, allowing heat on the workpiece surface to quickly dissipate into the uncut regions, preventing significant temperature buildup.

When the cutting depth reaches 25 μm, the temperature no longer decreases. At this point, the contact area increases significantly, generating more frictional heat than the surface can dissipate. Before reaching this depth, even though frictional heat was increasing, the small contact area allowed the limited thermal conductivity of the material to effectively dissipate the heat, keeping the temperature low. However, 25 μ m represents the critical depth of equilibrium between heat generation and dissipation rates, marking the key point of the material’s thermal diffusion ability. Beyond the 25 μm threshold, as the cutting depth increases, the rate of heat accumulation exceeds the rate of heat dissipation. This imbalance causes rapid heat buildup, and the surface temperature rises accordingly. Thus, 25 μm acts as a critical depth, marking a transition from a dissipation-dominated to a heat-generation-dominated regime. At this point, the heat generated in the cutting zone can no longer be effectively transferred to the surrounding areas through the limited thermal pathways, leading to the formation of a localized high-temperature zone.

When the cutting depth exceeds 25 μm, the temperature rise becomes more pronounced. As the depth increases, the arc length and contact area between the tool and workpiece expand, leading to a significant increase in cutting forces. This not only amplifies frictional heat generation but also intensifies plastic deformation in the cutting zone, further contributing to deformation-induced heat. These heat-generating effects accumulate rapidly, while the limited thermal conductivity of alumina bioceramics prevents effective dissipation of the excess heat, causing it to concentrate in the cutting region. Additionally, the increase in cutting depth results in a higher material removal rate, which further enhances energy conversion efficiency. A greater proportion of the mechanical energy is converted into heat, leading to a steep rise in the local temperature within the cutting zone. At this point, the workpiece’s heat dissipation capacity approaches saturation, and the dissipated heat can no longer offset the continuously increasing heat generation. As a result, heat accumulates on the surface, driving the temperature higher. More importantly, with deeper cuts, the cutting forces and material deformation become increasingly localized in the contact zone. This concentration of stress within the contact area further intensifies the heat generation, exacerbating the thermal effects.

#### 4.1.2 The influence of spindle speed on the temperature of alumina bioceramics

**Fig 2:**
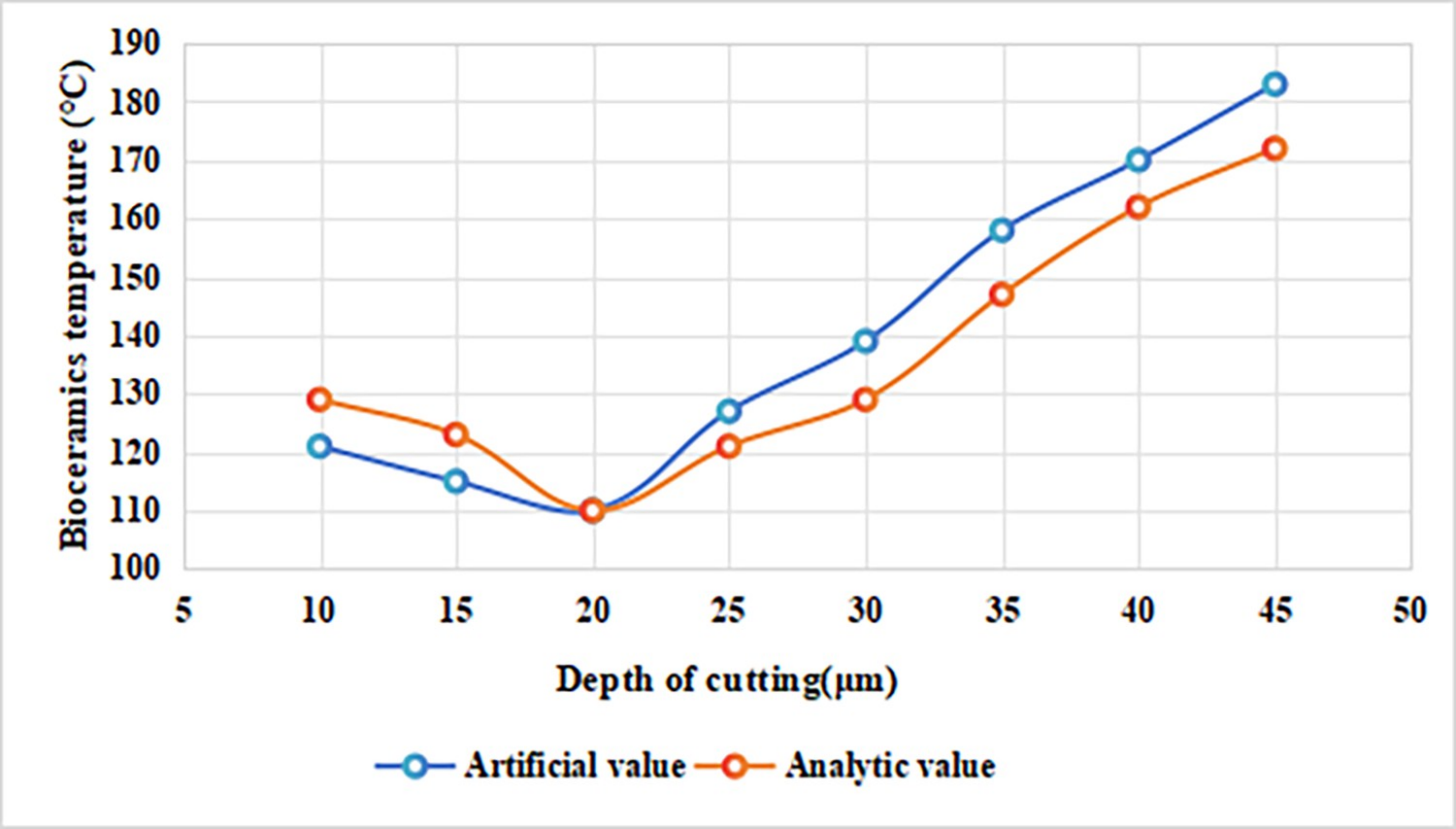
The effect of spindle speed on the maximum surface temperature of alumina bioceramics.

**Fig 3:**
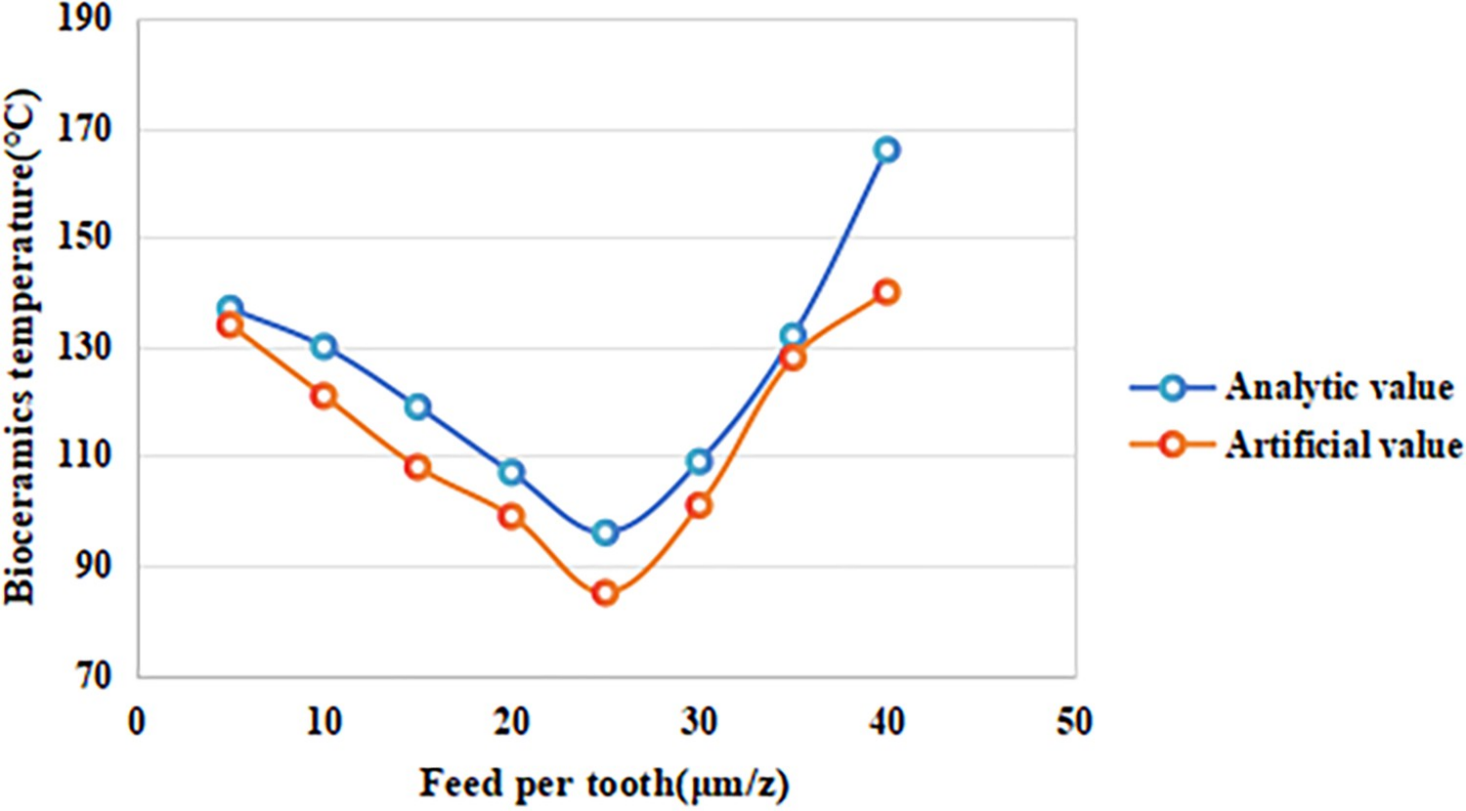
Effect of feed rate per tooth on the maximum surface temperature of alumina bioceramics.

In the micro-milling temperature field simulation, the cutting depth was set to 25 μm, with a feed per tooth of 25 μm/z, and spindle speeds of 12,000 r/min, 15,000 r/min, 18,000 r/min, 21,000 r/min, 24,000 r/min, 27,000 r/min, 30,000 r/min, and 33,000 r/min were selected to investigate the effect of spindle speed on the maximum surface temperature of alumina bioceramics. The results are shown in Fig 3. As shown in Fig 2, the theoretical analytical values and finite element simulation (FEM) values exhibit the same trend. However, the theoretical values are slightly higher than the simulated ones, likely due to simplifications made during the model construction. In the spindle speed range of 12,000 to 27,000 r/min, the surface temperature increases significantly with the spindle speed. This occurs because, as the spindle speed increases, the material removal rate per unit time also rises, resulting in more heat generation. The faster cutting process leads to a higher concentration of heat within the cutting zone, causing the surface temperature to rise. When the spindle speed exceeds 27,000 r/min, the temperature tends to stabilize or even decrease slightly. This phenomenon can be attributed to the reduced contact time between the tool and the bioceramic surface at higher spindle speeds. With shorter contact time, less heat accumulates at any given point on the workpiece. Additionally, the generated heat is more effectively distributed across the surface and dissipated into the surrounding material, preventing localized overheating.

#### 4.1.3 The effect of feed rate per tooth on the temperature of alumina bioceramics

**Fig 4:**
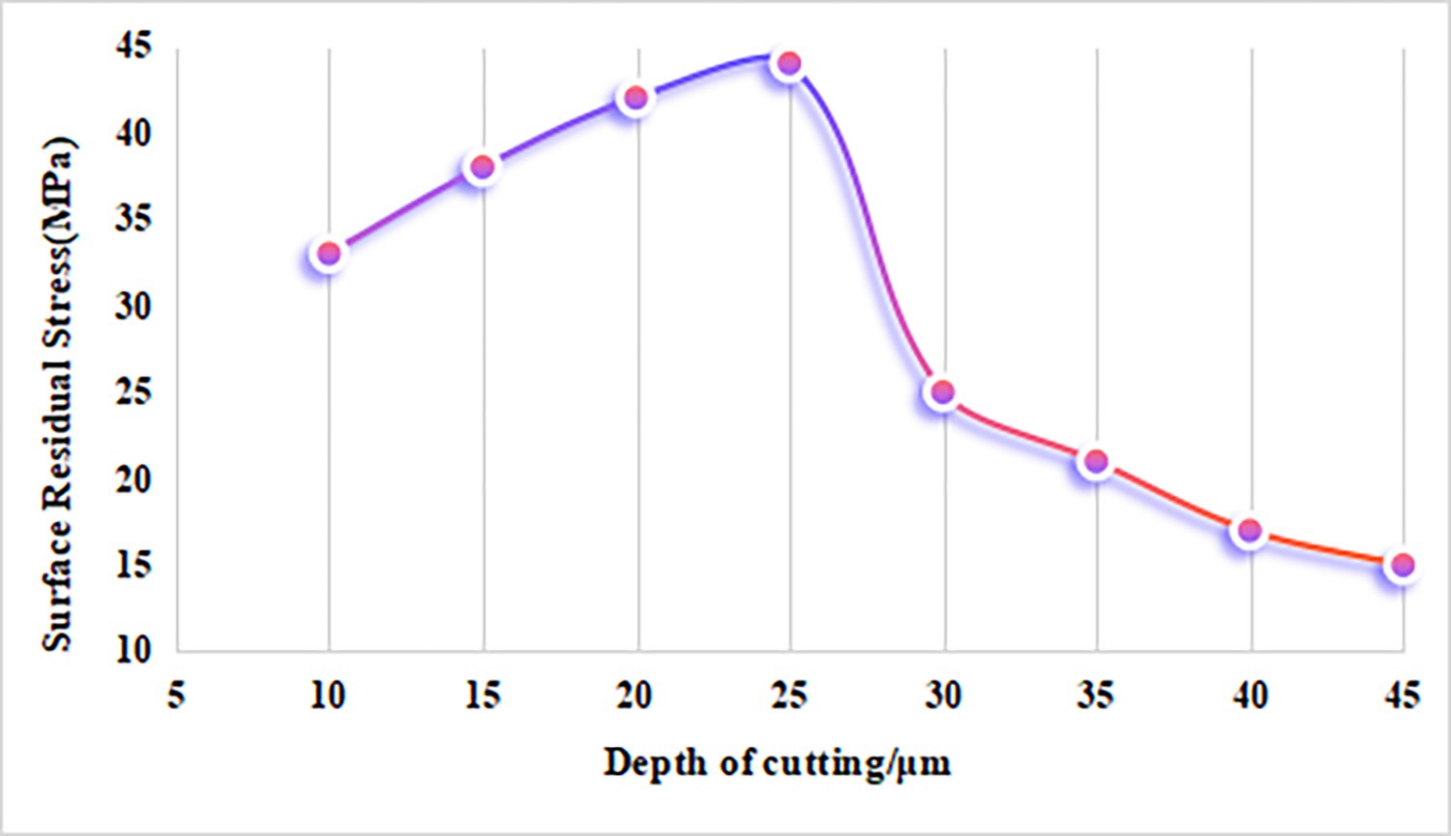
The effect of cutting depth on residual stress in alumina ceramics.

In the micro-milling temperature field simulation, the spindle speed was set to 18,000 r/min and the cutting depth to 25 μm, while the feed per tooth was varied at 5 μm/z, 10 μm/z, 15 μm/z, 20 μm/z, 25 μm/z, 30 μm/z, 35 μm/z, and 40 μm/z to explore the effect of feed rate on the maximum surface temperature of alumina bioceramics. The results are presented in Fig 4. As shown in Fig 3, both the analytical and simulation results demonstrate that the temperature exhibits a nonlinear trend, initially decreasing and then increasing as the feed per tooth increases. This behavior aligns with findings in the micro-machining of other brittle materials, such as zirconia and silicon nitride [31]. When the feed per tooth is less than 25 μm/z, the surface temperature decreases with increasing feed rate. This occurs because at lower feed rates: Cutting forces are smaller, reducing the contact area between the tool and the workpiece. Frictional heat generation is limited, resulting in slower temperature rise. The small amount of material removed per cut allows the tool to spend less time in contact with the surface, providing more opportunities for the generated heat to dissipate into the surrounding material, thereby preventing localized temperature accumulation. When the feed rate exceeds 25 μm/z, the surface temperature rises rapidly with increasing feed per tooth. This is due to several compounding factors: More material is removed per tooth, increasing the contact area between the tool and the workpiece, leading to higher cutting forces and more frictional heat. The rapid accumulation of heat cannot be effectively dissipated, given the poor thermal conductivity of alumina bioceramics, resulting in localized heat concentration in the cutting zone. Plastic deformation of the material intensifies as the feed rate increases, further contributing to heat generation and expanding the high-temperature region.

### 4.2 Analysis of residual stress simulation results

Finite element simulation can display the distribution and magnitude of residual stress on the surface and along the vertical direction of the workpiece. This study uses a spindle speed of 33,000 r/min, a cutting depth of 25 μm, and a feed per tooth of 25 μm/z. The residual stress distribution on the surface of alumina bioceramics is significantly non-uniform, with most areas exhibiting residual tensile stress. In the finite element model, the top and bottom surfaces of the tool are modeled as flat, meaning that during the simulation, the cutting edge presses and cuts the unmachined material through compression and friction. Stress concentration is observed in the areas where the tool edge contacts the workpiece, as these zones are the first to fracture, forming chips that are carried away by the tool. At the tool-material interface, cutting heat and compressive forces cause stress concentration. Due to the cutting and extrusion effects, localized stress accumulates where the ceramic material contacts the tool’s edge. After the cutting process, residual tensile or compressive stress forms on the surface and within the material.

#### 4.2.1 The influence of cutting depth on residual stress

As shown in Fig 4, when the cutting depth increases while other machining parameters remain constant, the residual stress on the workpiece surface first increases and then rapidly decreases. The milling simulation results indicate that residual tensile stress develops on the surface of alumina bioceramics after machining. When the cutting depth is less than 25 μm, the residual tensile stress increases with the increase in cutting depth. This is because the stress at shallow depths is primarily influenced by a combination of thermal stress and low mechanical stress, which facilitates crack initiation and propagation, leading to higher residual tensile stress on the surface. However, when the cutting depth exceeds 25 μm, the surface residual tensile stress decreases rapidly, indicating a rise in mechanical residual stress. As the cutting depth increases, the cracks extend beyond the surface layer into the material’s interior. The higher mechanical stress causes these cracks to propagate quickly, potentially resulting in larger-scale brittle spalling. This process not only releases surface residual stress but also alters the stress distribution pattern, as mechanical stress begins to have a more significant impact within the material. Consequently, brittle fractures in deeper regions shift the stress concentration away from the surface, reducing the residual tensile stress in the previously affected areas.

#### 4.2.2 The influence of spindle speed on residual stress

**Fig 5:**
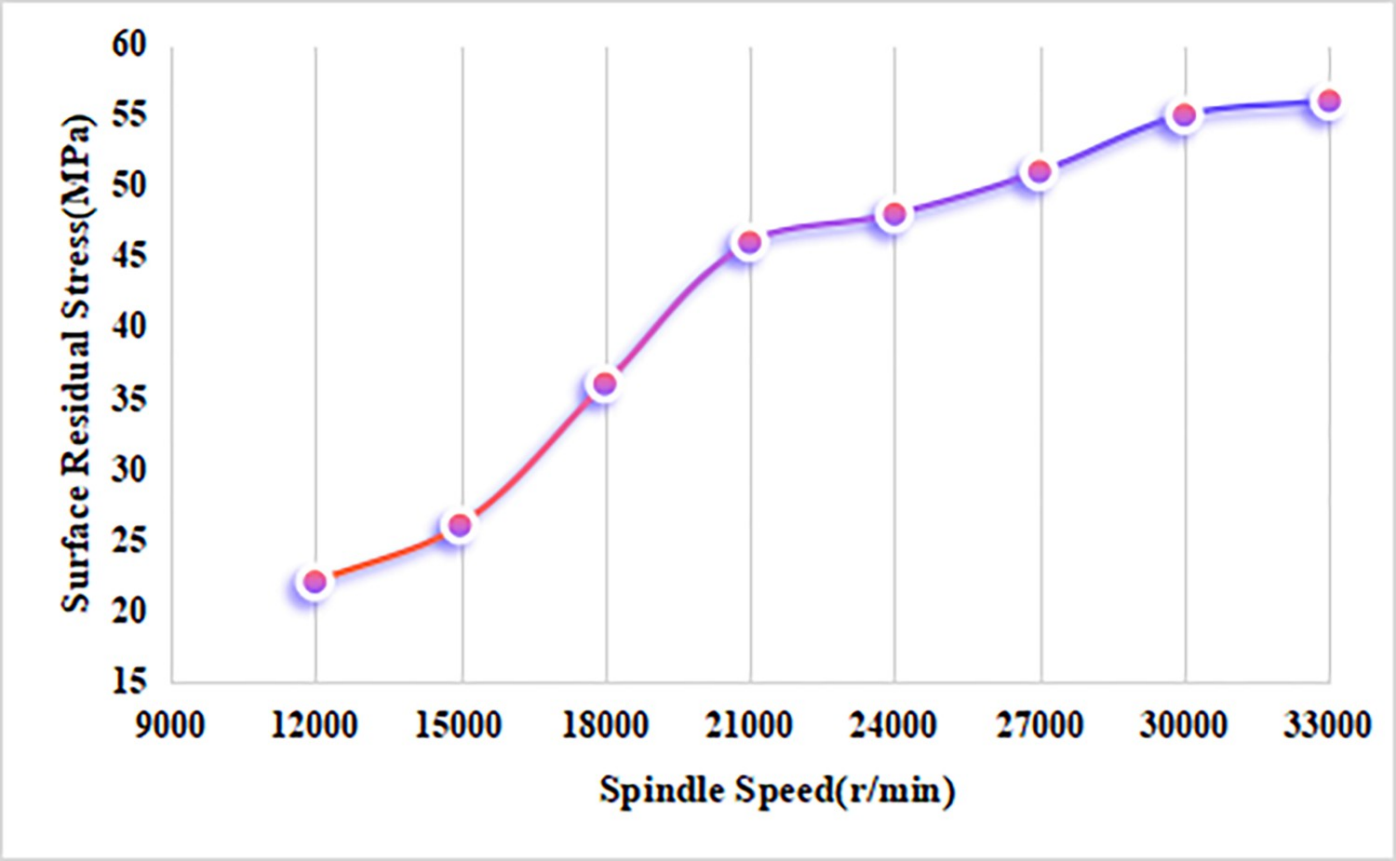
The effect of spindle speed on residual stress in alumina ceramics.

Fig 5 illustrates the effect of spindle speed on the residual stress in alumina bioceramics. It shows that, with other parameters held constant, the residual tensile stress on the ceramic surface increases as the spindle speed rises. The residual stress exhibits a trend of rapid initial increase, followed by a gradual leveling off after the spindle speed reaches 21,000 r/min. This behavior can be explained by the fact that, during micro-milling, an increase in spindle speed reduces the time per tool revolution, resulting in a higher cutting heat generation rate within a given time, which intensifies thermal stress on the ceramic surface. As discussed earlier, the cutting force decreases with increasing spindle speed, while the temperature rises rapidly, confirming the correlation between spindle speed and cutting temperature. Under thermo-mechanical coupling, the thermal stress induced by cutting heat becomes the primary source of stress as spindle speed increases, while the mechanical stress caused by tool pressure weakens. Consequently, the surface residual tensile stress in alumina bioceramics increases steadily under the combined effects of cutting force and cutting heat.

#### 4.2.3 The influence of feed rate per tooth on residual stress

**Fig 6:**
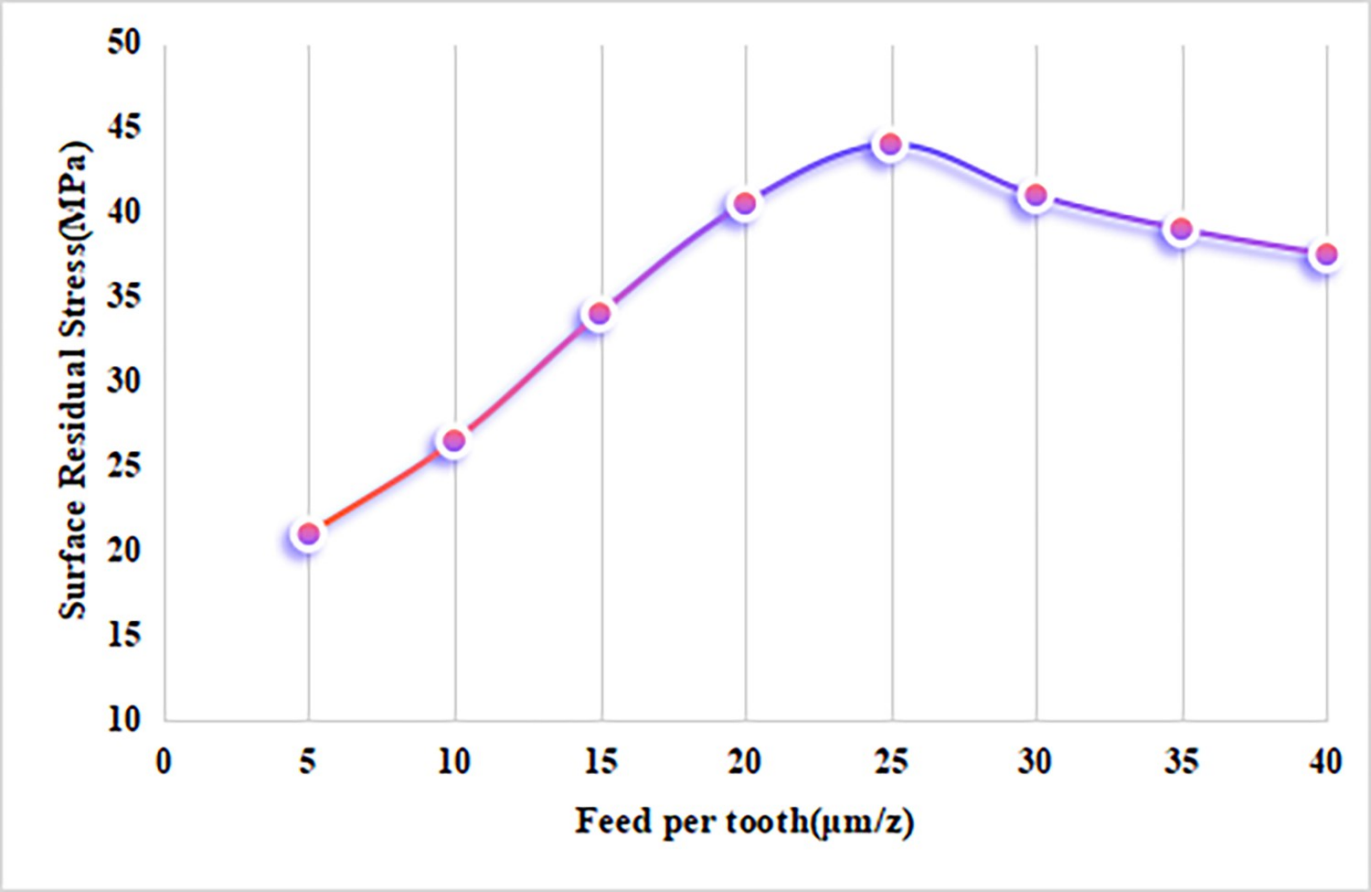
Effect of feed rate per tooth on residual stress in alumina ceramics.

As shown in Fig 6, with the spindle speed and cutting depth held constant, the residual tensile stress on the surface of alumina bioceramics first increases and then gradually decreases as the feed per tooth increases. When the feed per tooth is less than 25 μm/z, the residual tensile stress shows a significant upward trend. Analysis of the temperature variation curve with respect to the feed per tooth confirms that, at smaller feed rates, the workpiece temperature is higher while the cutting force is lower, as indicated by the simulation results. This combination contributes to the increase in surface residual tensile stress. However, when the feed per tooth exceeds 25 μm/z, the residual tensile stress starts to decrease. This suggests that plastic deformation effects on the ceramic material become more pronounced at higher feed rates, leading to an increase in mechanical residual stress. The shift from thermal stress dominance to mechanical stress explains the observed reduction in surface residual tensile stress at higher feed rates.

## 5 Discussion

This study systematically investigated the effect of micro-milling parameters on the surface temperature and residual stress of alumina bioceramics through three-dimensional finite element simulation. A milling tool with a length of 2 mm, diameter of 1 mm, and thickness of 0.2 mm was used, along with a workpiece model measuring 3 mm × 2 mm × 1 mm. The milling temperature field model and residual stress simulation model were constructed to examine the influence of cutting depth, spindle speed, and feed per tooth on the surface temperature and residual stress of alumina bioceramics. The results showed that when the cutting depth, spindle speed, and feed per tooth were set to 25 μm, 21,000 r/min, and 25 μm/z, respectively, the surface temperature and residual stress reached optimal levels, which helps enhance the material’s crack resistance and service life.

This study builds on previous research by numerous scholars and introduces innovations and expansions in the analysis of micro-milling parameters affecting the residual stress in alumina bioceramics. Dai et al. [32] investigated the effect of cutting depth on the surface integrity of ceramic materials, finding that cutting depth significantly influences surface roughness and microhardness, but they did not explore the residual stress patterns in detail. In contrast, this study provides a systematic analysis of the cutting depth, spindle speed, and feed per tooth, offering a more comprehensive understanding of their combined effects on both surface temperature and residual stress. This systematic approach provides a more thorough theoretical basis for optimizing micro-milling parameters. Wambua et al. [33] experimentally studied the effect of cutting speed and feed rate on cutting force and temperature in alumina ceramics, demonstrating that high cutting speeds significantly increase cutting temperature. This finding aligns with the results of the present study, further confirming the significant impact of high speeds on temperature. However, Wambua’s study did not address residual stress. In comparison, this study utilizes three-dimensional finite element simulation, enhancing the precision of the results and providing a deeper exploration of how different parameters affect the mechanism of residual stress formation. Xu et al. [34] examined the impact of micro-milling on surface damage in ceramics and proposed methods for optimizing cutting parameters. However, their research focused primarily on surface damage and roughness, lacking a systematic analysis of residual stress. In contrast, the present study delves into the influence of key parameters on residual stress, offering more comprehensive theoretical support for the optimization of micro-milling processes.

The main contributions of this study are as follows: First, systematic analysis of the effects of micro-milling parameters on the surface temperature and residual stress of alumina bioceramics using finite element simulation. This addresses the gap in existing research regarding the analysis of residual stress. Secondly, theoretical support for optimizing micro-milling parameters by investigating key factors such as cutting depth, spindle speed, and feed per tooth, contributing to the improvement of alumina bioceramics’ machining quality. Thirdly, broader applicability: The methods and conclusions of this study are not limited to alumina bioceramics but can also be extended to the micro-milling of other hard-to-machine materials, offering significant engineering application value. However, this study has certain limitations. The long-term effects of residual stress are crucial in practical applications, but this study does not explore the temporal evolution of residual stress. Furthermore, the analysis relies primarily on finite element simulation, with limited experimental validation. Although the simulation results align with some theoretical analytical values, further experimental calibration and validation of the simulation model are necessary to ensure the reliability and accuracy of the results.

## 6 Conclusion

This study systematically investigated the effects of micro-milling parameters on the surface temperature and residual stress of alumina bioceramics using three-dimensional finite element simulation, achieving the following key findings:

At smaller cutting depths, the surface temperature decreases as depth increases, but it rises significantly when the depth exceeds 25 μm. In the spindle speed range of 12,000 r/min to 27,000 r/min, the temperature increases with speed, but beyond 27,000 r/min, it stabilizes or slightly decreases. When the feed per tooth is low, temperature decreases as the feed rate increases, but beyond 25 μm/z, the temperature rises sharply.

The residual stress simulation results show that the variation patterns of residual stress align with the temperature trends, reflecting the thermo-mechanical coupling effect on residual stress. At smaller cutting depths, the residual tensile stress increases with depth but decreases rapidly beyond 25 μm. The residual tensile stress peaks at a spindle speed of 21,000 r/min and becomes stable at higher speeds. The residual tensile stress is highest at a feed rate of 25 μm/z, but it gradually decreases as the feed rate exceeds this value.

This study provides a scientific basis for optimizing the micro-milling process through the investigation of multiple machining parameters. Future research could further explore the evolution of residual stress over time to understand its long-term effects. Additionally, experimental validation of the simulation results is necessary to ensure the reliability and accuracy of the conclusions. Other micro-milling parameters, such as the use of coolants and different milling paths, could also be considered in future studies to further optimize the machining process of alumina bioceramics.

## Supporting information

S1 Data (XLSX)

S2 Data (XLSX)

S3 Data (XLSX)

S4 Data (XLSX)

S5 Data (XLSX)

S6 Data (XLSX)
